# Validation of a New Patient-Reported Outcome Measure of the Functional Impact of Essential Tremor on Activities of Daily Living

**DOI:** 10.5334/tohm.886

**Published:** 2024-05-14

**Authors:** Ludy C. Shih, Michael T. Stevenson, Steven Bellows, Alfonso Fasano, Sheng-Han Kuo, Kelly E. Lyons, Henry Moore, Holly A. Shill, Aparna Wagle Shukla, Carlos Singer, Rodger J. Elble

**Affiliations:** 1 Beth Israel Deaconess Medical Center, Department of Neurology, Boston, MA 02215, US; 2 Harvard Medical School, Boston, MA 02215, US; 3 Boston Medical Center, Department of Neurology, Boston, MA 02118, US; 4 Baylor College of Medicine, Department of Neurology, Houston, TX, US; 5 Edmond J. Safra Program in Parkinson’s Disease, Morton and Gloria Shulman Movement Disorders Clinic, Toronto Western Hospital, UHN, Toronto, Ontario, Canada; 6 Division of Neurology, University of Toronto, Toronto, Ontario, Canada; 7 Krembil Brain Institute, Toronto, Ontario, Canada; 8 Columbia University, Department of Neurology, New York, NY, US; 9 University of Kansas Medical Center, Department of Neurology, US; 10 University of Miami School of Medicine, Department of Neurology, Miami, FL, US; 11 Barrow Neurological Institute, Phoenix, AZ, US; 12 Fixel Institute for Neurological Diseases, University of Florida, Department of Neurology, Gainesville, FL, US; 13 Southern Illinois University School of Medicine, Department of Neurology, Springfield, IL, US

**Keywords:** essential tremor, patient-reported outcome measure, depression, anxiety, coping skills

## Abstract

**Background:**

The Essential Tremor Rating Assessment Scale (TETRAS) is a popular scale for essential tremor (ET), but its activities of daily living (ADL) and performance (P) subscales are based on a structured interview and physical exam. No patient-reported outcome (PRO) scale for ET has been developed according to US regulatory guidelines.

**Objective:**

Develop and validate a TETRAS PRO subscale.

**Methods:**

Fourteen items, rated 0–4, were derived from TETRAS ADL and structured cognitive interviews of 18 ET patients. Convergent validity analyses of TETRAS PRO versus TETRAS ADL, TETRAS-P, and the Quality of Life in Essential Tremor Questionnaire (QUEST) were computed for 67 adults with ET or ET plus. Test-retest reliability was computed at intervals of 1 and 30 days. The influence of mood (Hospital Anxiety and Depression Scale, HADS) and coping behaviors (Essen Coping Questionnaire, ECQ) was examined with multiple linear regression.

**Results:**

TETRAS PRO was strongly correlated (r > 0.7) with TETRAS ADL, TETRAS-P, and QUEST and exhibited good to excellent reliability (Cronbach alpha 95%CI = 0.853–0.926; 30-day test-retest intraclass correlation 95%CI = 0.814–0.921). The 30-day estimate of minimum detectable change (MDC) was 6.6 (95%CI 5.2–8.0). TETRAS-P (r_semipartial_ = 0.607), HADS depression (r_semipartial_ = 0.384), and the coping strategy of information seeking and exchange of experiences (r_semipartial_ = 0.176) contributed statistically to TETRAS PRO in a multiple linear regression (R^2^ = 0.67).

**Conclusions:**

TETRAS PRO is a valid and reliable scale that is influenced strongly by tremor severity, moderately by mood (depression), and minimally by coping skills. The MDC for TETRAS PRO is probably sufficient to detect clinically important change.

## Introduction

Essential tremor (ET) is the most common form of pathologic tremor. A recent consensus statement defined ET as an isolated tremor syndrome of bilateral upper limb action tremor of at least 3 years duration, with or without tremor in other locations (e.g., head, voice, lower limbs) and with no other neurological signs, such as dystonia, ataxia, or parkinsonism [1], and this definition is used in this report. The same consensus statement defined ET plus as tremor with the characteristics of ET but with “additional neurological signs of uncertain significance such as impaired tandem gait, questionable dystonic posturing, memory impairment or other mild neurologic signs of unknown significance that do not suffice to make an additional syndrome classification or diagnosis.” In other words, ET plus is a documentation of diagnostic uncertainty in a patient who otherwise exhibits the characteristics of ET.

The Essential Tremor Rating Assessment Scale (TETRAS) [2] has become a standard for the clinical assessment of ET, due to its excellent clinimetrics and extensive validation [3,4,5]. TETRAS was developed by the Tremor Research Group specifically for the clinical assessment of ET. TETRAS consists of two subscales: 1) an interview-based assessment of activities of daily living (ADL) and 2) a performance-based assessment (TETRAS-P) of tremor amplitude.

Patient reported outcome measures (PROMs) are reports of any aspect of a patient’s health status that come directly from the patient and can encompass measurement of symptoms, ADLs, and quality of life [6]PROMs are recommended by the United States Food and Drug Administration (FDA) in the clinical development of new investigational drugs and devices in order to provide evidence of treatment benefit from a patient’s perspective [6]. Functional impact of tremor on activities of daily living is an important topic of concern for ET patients, and we chose to focus a PROM on this concept of interest. An existing patient-reported tremor questionnaire for ADLs has 25 four-point Likert items [7], but this scale was not developed and validated according to current best practices [8] and FDA guidelines [6]. The TETRAS ADL and performance subscales are now widely used in clinical trials, but there is no TETRAS PROM. TETRAS PRO was designed to measure the impact of tremor on common activities of daily living that are known to be affected by ET. The intended context of use for TETRAS PRO is the same as for TETRAS ADL and TETRAS-P: the assessment of tremor severity and its impact on ADLs in the clinic and in clinical trials. We now report the development and validation of TETRAS PRO.

## Methods

The protocol and consent were approved by the Southern Illinois University School of Medicine Institutional Review Board (IRB) and by the local IRB of each participating clinical site. All participants were recruited in accordance with their local IRB using a standardized protocol. Our procedures of PROM development were in accordance with the COSMIN guideline for studies of measurement properties of patient reported outcome measures [9].

### Initial development of TETRAS PRO

The first version or prototype of TETRAS PRO was derived from the 12 items of the clinician-administered TETRAS ADL, which was designed to assess the functional impact of tremor on activities of daily living that are known to be affected by ET. Two additional items addressing the impact of head tremor and lower extremity tremor were added to make TETRAS PRO more comprehensive and in line with TETRAS-P. Each item had 5 response choices, rated 0–4, that were carefully worded to be clearly distinguishable in severity and to be compatible with eighth-grade reading skills.

Content validity and acceptability of the initial version of TETRAS PRO were explored with structured cognitive interviews of 18 adult patients with ET or ET plus. These patients were examined at one of two sites with TETRAS ADL and TETRAS-P during a routine clinic visit for ET. Following the clinic visit, the patients completed TETRAS PRO and were then interviewed by a trained interviewer. Each interview was audio recorded without patient identification, as recommended by the FDA. The patients were asked to read each TETRAS PRO item out loud and answer three additional questions to determine 1) if the question is clear, 2) if the five choices are clear and appropriate, and 3) if the question addresses an activity that is important to the patient. At the end of the interview, patients were also asked if any activities or issues of importance were not addressed in the TETRAS PRO.

Data from the structured interviews dictated some rewording of the 14 items but no substantive changes. TETRAS PRO version 2 was then given to the other movement disorder specialists in this study (N = 8) for their comments on relevance and comprehensiveness. This resulted in some changes in item wording but no addition or deletion of items. The validity and reliability of TETRAS PRO version 3 (Supplementary Material 1) were then studied as follows.

### Multicenter scale validation study

Nine participating sites across the US and Canada used the same protocol, inclusion and exclusion criteria, assessments, and case report forms. Inclusion criteria were based on the consensus criteria of the International Parkinson and Movement Disorder Society for ET and ET plus [2]. These diagnoses were based on the clinician’s exam and judgment. The examining clinicians were specifically asked to document the presence or absence of unsteady tandem gait, body posturing that could be dystonic, questionable rest tremor, definite rest tremor, jerky tremor, peripheral neuropathy, and mild cognitive impairment. A standardized exam with specific criteria for distinguishing ET from ET plus was not used because none exists. All patients were age 18 or older and exhibited bilateral upper limb action tremor of at least 3 years duration, with or without tremor in other locations (e.g., head, voice, or lower limbs). Patients were excluded if they had prior surgery for tremor (e.g., deep brain stimulation or thalamotomy) or any medical illness or cognitive impairment that would interfere with the assessment of tremor. Patients with other tremor syndromes like dystonia with tremor, Parkinson disease, focal tremor of the voice or head, orthostatic tremor, and task-specific or position-specific tremor were excluded.

Participants were screened and consented in person on day 0. The in-clinic assessment consisted of the TETRAS ADL and TETRAS-P subscales [3], Essen Coping Questionnaire (ECQ) [10], Hospital Anxiety and Depression Scale (HADS) [11], and Quality of Life in Essential Tremor Questionnaire (QUEST) [12].

TETRAS ADL requires a clinician interview to elicit the impact of tremor on the 12 ADLs of speaking, feeding, drinking, hygiene, dressing, pouring, carrying objects, using keys, writing, working, social impact, and the activity affected by tremor that is most important to the patient. TETRAS-P is a clinician-administered examination consisting of 9 items to assess tremor severity (amplitude) in the head, face, voice, upper and lower limbs and during writing and spiral drawing tasks.

The ECQ [10] (www.psychometrikon.de) was designed to assess coping efforts in patients with chronic disease [10]. The ECQ has nine subscales each with five questions that are scored on a 0–4 Likert scale (not at all, somewhat, moderately, strongly, or extremely). Each raw subscore is converted to a 1–9 range of integer stanine scores using the conversion scheme in the ECQ manual (www.psychometrikon.de) [10]. The nine subscales of ECQ are 1) acting and problem-oriented coping (APC), 2) distance and self-promotion (DSP), 3) information seeking and exchange of experiences (ISE), 4) trivialization, wishful thinking and defense (TWD), 5) depressive processing (DP), 6) willingness to accept help (WAH), 7) active search for social integration (ASS), 8) trust in medical care (TMC), and 9) finding of inner stability (FIS).

The HADS has two subscales: anxiety and depression [11]. Each subscale consists of seven questions with 4 response choices scored 0–3. The maximum HADS anxiety (HADS-A) and depression (HADS-D) scores are 21. The subscale and total scores are suitable for patients with relatively mild symptoms of anxiety and depression [11,13,14,15].

The Quality of Life in Essential Tremor Questionnaire (QUEST) is a 30-item Likert scale with 0–4 ratings (maximum score 120) and was developed specifically as a PROM for assessing the impact of ET on quality of life [12]. The 30 items are parceled into 5 subscales (communication, hobbies and leisure, psychosocial, work and finances, and physical). Each subscale score is expressed as a percentage of the maximum possible score. QUEST has been used extensively in studies of ET [5].

TETRAS PRO is a 14-item questionnaire that addresses ADLs affected by ET. Each item consists of a question pertaining to an activity that is likely to be affected by ET (Table 1), followed by 5 ordinal responses on a 0 to 4 scale (Supplementary Material 1).

**Table 1:** The 14 items of TETRAS PRO.


**Instructions:** For each question, **circle or mark the answer that best describes your typical condition during the past week**. A family member or friend can circle your answers if you cannot do this yourself. However, we want you to answer each question without discussing the answers with other people.

1. Is your voice shaky or tremulous?

2. Does your head shake?

3. Does tremor affect your ability to eat soup or other liquids with a regular soup spoon?

4. Does tremor affect your ability to drink liquids from a regular 8-ounce (250 ml) glass or cup?

5. Does tremor interfere with your hygiene or personal care (bathing, shaving, brushing teeth, using makeup)?

6. Does tremor interfere with your ability to dress yourself? This includes putting on jewelry, tying shoelaces, zipping zippers, and managing small buttons.

7. Does tremor affect your ability to pour liquids from a carton, bottle or pitcher?

8. Does tremor affect your ability to pass food or drink to someone at your dinner table?

9. Does tremor limit your ability to use a keypad on a phone, lock or computer?

10. Does tremor affect your ability to write?

11. Does tremor affect your job performance? This would include being a homemaker. If you are retired, consider how your current tremor would have affected your previous job.

12. Do you notice tremor in your legs?

13. Think about how tremor affects your daily activities. In the space below, please write the activity that is most important to you. Activity _______________________________. How much does your tremor interfere with this activity?

14. Has tremor affected your social activities?


TETRAS PRO, TETRAS ADL, TETRAS-P, QUEST, HADS, and ECQ were completed in clinic. Participants were given two copies of the TETRAS PRO with return envelopes, clearly marked to be completed at home the next day (day 1) and one month later (day 30) and returned to each site by mail upon completion. The two at-home TETRAS PRO assessments were used to estimate test-retest reliability. Data from patients that received any new investigational or conventional treatment or any change in dosing regimen during the study period were excluded from the test-retest reliability analyses.

### Statistical Analyses

As a rule, 100 subjects are desirable for estimating convergent validity of a PROM [16]. However, the proposed PROM for ET was designed to be a unidimensional construct, and based on our prior experience with TETRAS ADL, we were confident that fewer than 100 patients would be adequate. The first 17 patients were enrolled at Southern Illinois University. A preliminary analysis of data from these patients revealed a Cronbach alpha of 0.937 (95% lower confidence limit 0.893) for TETRAS PRO, and the elimination of no item changed this value more than ± 0.01. Test-retest reliability of TETRAS PRO was 0.869 (95% confidence limits 0.672–0.952). Using the on-line calculator of Arifin [17], a sample size of 63 is adequate for a Cronbach alpha of 0.8, α = 0.05, power = 80%, and expected dropout or missing data rate of 10%. This sample size would also be more than adequate for estimating the test-retest reliability (two-way random-effects, absolute-agreement intraclass correlation): 55 patients are adequate for an assumed test-retest reliability of 0.8. Therefore, we were confident that a sample size of 70 patients, providing a subject-to-item ratio of 5, would be sufficient for scale validation.

All data were stored in a REDCap database (www.project-redcap.org) administered and supervised by the lead site and investigators at Boston University School of Medicine. Case report forms were reviewed for missing data, and the database was reviewed for accuracy prior to statistical analyses. Statistical analyses were performed using MedCalc**®** Statistical Software version 22.016 (www.medcalc.org) and JASP version 0.18.2 (www.jasp-stats.org). Convergent validity of TETRAS PRO was assessed by computing the linear correlation of day 0 TETRAS PRO scores with TETRAS ADL, TETRAS-P, and QUEST. Test-retest reliability of TETRAS PRO was assessed by intraclass correlation (two-way random effects model, absolute agreement ICC). Internal consistency among the TETRAS PRO items was assessed with Cronbach alpha and McDonald omega. Multiple linear regression was used to examine contributions of HADS and ECQ to TETRAS PRO, TETRAS ADL, and TETRAS-P variance. We hypothesized that HADS and ECQ would have no correlation with TETRAS-P because TETRAS-P is an examiner’s appraisal of tremor severity. In contrast, we hypothesized that TETRAS PRO and TETRAS ADL would have some correlation with HADS and possibly ECQ, and strong correlation with TETRAS-P. A multiple linear regression model was used to assess the impact of TETRAS-P, HADS and ECQ on TETRAS ADL and on TETRAS PRO: TETRAS PRO (or TETRAS ADL) = β0 + β1**·**TETRAS-P + β2**·**HADS + β3**·**ECQ. All independent variables were entered simultaneously (forced entry method) in all multiple linear regression analyses. Pearson correlations were interpreted as follows: ≤0.39 = weak correlation; 0.4–0.69 = moderate correlation; 0.7–0.89 = strong correlation; ≥0.9 = very strong correlation. The interpretations of intraclass correlations were poor for <0.5, moderate for 0.5–0.75, good for 0.76–0.9, excellent for >0.9. Minimum detectable change was estimated from Bland-Altman plots (±1.96 times the standard deviation of the differences) [18,19]. Exploratory factor analysis of TETRAS PRO was performed.

## Results

### Cognitive interviews

Patient responses in the cognitive interviews are summarized in Supplementary Material 2. TETRAS PRO item 1 (Is your voice shaky or tremulous?), item 2 (Does your head shake?), and item 12 (Do you notice tremor in your legs?) were viewed as unimportant by 10, 8, and 12 of 18 patients, respectively. Table 1 in Supplementary Material 2 summarizes the distributions of TETRAS PRO ratings for each item and the YES/NO classification of importance. Factors other than perceived functional impairment appeared to influence the patients’ classification of items as important or not. We did not systematically search for these factors, but patient comments included fear of future development, acceptance of tremor, ability to ignore tremor, and tolerable changes in lifestyle (e.g., stress avoidance). Therefore, we retained these items because they reflect known features of ET and ET plus (i.e., had face validity), and we wanted to explore the validity of these items vis-à-vis clinician ratings in TETRAS-P.

### Validity and internal consistency analyses

#### Patient cohort for the validity analysis

Data from 26 of 93 patients were excluded from the validity analysis because these patients were inadvertently given the wrong (i.e., older) version of TETRAS PRO on day 0. Thirty-seven men (55%) and 30 women (45%) completed the correct version of TETRAS PRO on day 0. Diagnoses were ET in 58 (87%) and ET plus in 9 (13%). The reasons for the ET plus classification were unsteady tandem gait (n = 5), body posturing that could be dystonic (n = 3), mild cognitive impairment (n = 3), and rest tremor (n = 2) (see Supplementary Material 4). One patient identified as Asian (1.5%), two identified as Black (3.0%), and the remainder identified as White. Mean patient age, age of onset, and duration of tremor were 68.2 (SD 10.0, range 31.8–82.6), 39.9 (SD 19.7, range 5.0–74.0), and 28.3 (SD 18.8, range 3.8–67.4) years. Only 2 patients had <12 years of education (6 and 11 years). Mean (SD) TETRAS ADL, TETRAS-P, and TETRAS PRO scores were 24.3 (7.2), 22.8 (6.9), and 22.9 (9.2). All three measures did not deviate significantly from a normal distribution (Shapiro-Wilk test).

#### Internal consistency of TETRAS PRO

TETRAS PRO had good internal consistency across the 14 items, with a Cronbach alpha of 0.894 (95% confidence limits 0.853–0.926). Dropping each of the individual 14 items did not reduce Cronbach alpha below 0.879 or increase it above 0.909. Item-rest correlations exceeded 0.5 except for item 1 voice (0.386), item 2 head (0.064), and item 12 legs (0.356). The deletion of these three items resulted in a Cronbach alpha of 0.916 (95% confidence limits 0.882–0.942). McDonald omega values were essentially identical to Cronbach alpha: McDonald ω = 0.903 (95% CI = 0.869–0.937). Head tremor item 2 was the only item with an item-rest correlation less than 0.3 (Supplementary Material 3), and exploratory factor analysis and scree plot were consistent with a single factor construct for TETRAS PRO (Supplementary Material 3).

Item 1 voice, item 2 head, and item 12 leg were viewed unimportant by 10, 8 and 12 of 18 patients who participated in the cognitive interviews (Supplementary Material 2). We therefore examined the validity of these items by correlating each with the corresponding item in TETRAS-P. Spearman’s rho for the TETRAS PRO item versus the corresponding TETRAS-P item was 0.594 (p < 0.001) for voice, 0.588 (p < 0.001) for head, and 0.186 (p = 0.074) for leg tremor.

#### Correlation of TETRAS PRO with TETRAS-P and TETRAS ADL

**Figure 1:**
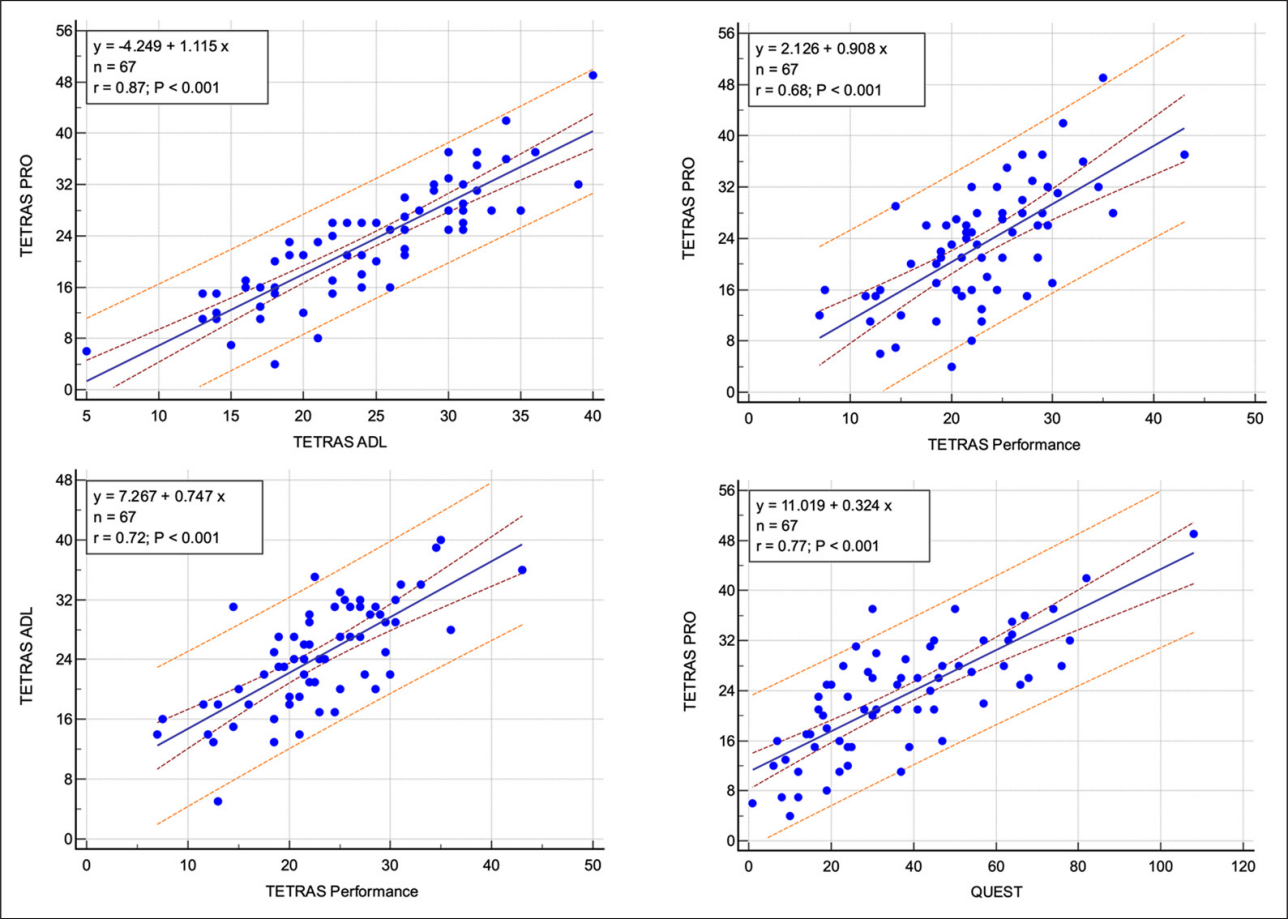
**Linear regression of TETRAS PRO with TETRAS ADL, TETRAS Performance, and QUEST**. A linear regression of TETRAS ADL vs TETRAS Performance is shown for comparison. The 95% confidence limits (maroon) and 95% prediction limits (orange) are shown. The maximum possible scores of TETRAS PRO, TETRAS ADL, AND TETRAS Performance are 56, 48, and 64.

TETRAS PRO had a strong linear relationship with TETRAS ADL and TETRAS-P (Figure 1). The correlation between TETRAS ADL and TETRAS-P did not differ significantly from the correlation between TETRAS PRO and TETRAS-P (z statistic = 0.444, p = 0.66) (Figure 1).

#### Correlation of TETRAS PRO with QUEST

The mean total QUEST score was 36.5 (SD 22.0, range 1–108) and was positively skewed (median = 31). TETRAS PRO was strongly correlated with QUEST total score (r = 0.77, p < 0.01; Figure 1). Multiple linear regression of TETRAS PRO (dependent variable) versus the QUEST subscales revealed a statistically significant contribution from QUEST physical (r = 0.709, p < 0.0001) and work/finances (r = 0.323, p < 0.01). The same model with TETRAS ADL as the dependent variable revealed a significant correlation only with QUEST physical (r = 0.710, p < 0.0001). Similarly, QUEST physical (r = 0.521, p < 0.0001) was the only significant correlate for TETRAS-P as the dependent variable.

#### Correlation of TETRAS subscales and QUEST with HADS

The mean total HADS score was 8.2 (SD 6.4, range 0–33) and was positively skewed (median = 7.0). Mean HADS-A was 4.8 (SD 3.9, range 0–16, median 4), and mean HADS-D was 3.4 (SD 3.2, range 0–17, median = 3). TETRAS PRO correlated significantly with HADS-A (r = 0.295, p = 0.0155) and HADS-D (r = 0.478, p < 0.0001), and HADS-A and HADS-D correlated significantly with each other (r = 0.631, p < 0.0001). Multiple linear regression of TETRAS PRO (dependent variable) versus HADS-A and HADS-D (predictor variables) revealed a statistically significant contribution from HADS-D (r_semipartial_ = 0.377, p = 0.0011) but not from HADS-A (r_semipartial_ = 0.0093, p = 0.9331). Similarly, multiple linear regression of TETRAS ADL score versus HADS-A and HADS-D revealed a statistically significant contribution from HADS-D (r_semipartial_ = 0.291, p = 0.015) but not from HADS-A (r_semipartial_ = 0.0144, p = 0.9019).

QUEST total score, by comparison, was correlated with HADS-A (r = 0.569, p < 0.0001) and HADS-D (r = 0.608, p < 0.0001). Multiple linear regression of QUEST total score versus HADS-A and HADS-D revealed a statistically significant contribution from HADS-D (r_semipartial_ = 0.320, p = 0.0012) and from HADS-A (r_semipartial_ = 0.239, p = 0.014).

#### Correlation of TETRAS PRO with ECQ

ISE (information seeking and exchange of experiences) was the only ECQ subscale with a statistically significant Pearson correlation with TETRAS PRO (r = 0.363, p = 0.0025). All other correlations were ≤ 0.19 (p > 0.12). ECQ-ISE also had the only statistically significant correlation with TETRAS ADL (r = 0.302, p = 0.013). By contrast, TETRAS-P did not correlate significantly with ECQ-ISE (r = 0.165, p = 0.183) or any other ECQ subscale.

#### Multiple regression of TETRAS PRO with TETRAS-P, HADS-D and ECQ-ISE

Based on the preceding analyses, we examined the following linear relationship: TETRAS PRO = β0 + β1**·**TETRAS-P + β2**·**HADS-D + β3**·**ECQ-ISE. The results are shown in Table 2. The coefficient of determination for this relationship was 0.672. The residual variance was normally distributed, and the variance inflation factors (VIF) for the three predictor variables were nearly 1, indicating no multicollinearity among predictor variables. A similar relationship was found for TETRAS ADL, but with no statistically significant contribution from ECQ-ISE (Table 3).

**Table 2:** Statistical relationship of TETRAS PRO with HADS-D, ECQ-ISE and TETRAS Performance.


LEAST SQUARES MULTIPLE REGRESSION							

Sample size	67						

Coefficient of determination R^2^	0.6715						

R^2^-adjusted	0.6559						

Multiple correlation coefficient	0.8195						

Residual standard deviation	5.4008						

**REGRESSION EQUATION**							

**Predictor variables**	**Coefficient**	**Std. Error**	**t**	**P**	**r**_partial_	**r**_semipartial_	**VIF**

(Constant)	–3.6996						

TETRAS-P	0.8267	0.09829	8.411	<0.0001	0.7273	0.6073	1.030

HADS-D	1.1361	0.2134	5.324	<0.0001	0.5571	0.3844	1.045

ECQ-ISE	0.8436	0.3458	2.439	0.0175	0.2938	0.1761	1.068

**ANALYSIS OF VARIANCE**							

**Source**	**DF**	**Sum of Squares**	**Mean Square**				

Regression	3	3756.8544	1252.2848				

Residual	63	1837.6531	29.1691				

F-ratio	42.9319						

Significance level	P < 0.0001						

**RESIDUALS**							

Shapiro-Wilk test for Normal distribution	W = 0.9874 accept Normality (P = 0.7367)						


**TETRAS PRO = β0 + β1·TETRAS-P + β2·HADS-D + β3·ECQ-ISE**.

**Table 3:** Statistical relationship of TETRAS ADL with HADS-D, ECQ-ISE and TETRAS Performance.


LEAST SQUARES MULTIPLE REGRESSION							

Method	Enter						

Sample size	67						

Coefficient of determination R^2^	0.6263						

R^2^-adjusted	0.6085						

Multiple correlation coefficient	0.7914						

Residual standard deviation	4.4741						

**REGRESSION EQUATION**							

**Independent variables**	**Coefficient**	**Std. Error**	**t**	**P**	**r**_partial_	**r**_semipartial_	**VIF**

(Constant)	3.9741						

TETRAS-P	0.7009	0.08142	8.608	<0.0001	0.7352	0.6630	1.030

HADS-D	0.6380	0.1768	3.609	0.0006	0.4140	0.2780	1.045

ECQ-ISE	0.4800	0.2865	1.676	0.0988	0.2066	0.1291	1.068

**ANALYSIS OF VARIANCE**							

**Source**	**DF**	**Sum of Squares**	**Mean Square**				

Regression	3	2113.3264	704.4421				

Residual	63	1261.0915	20.0173				

F-ratio	35.1916						

Significance level	P < 0.0001						

**RESIDUALS**							

Shapiro-Wilk test for Normal distribution	W = 0.9912 accept Normality (P = 0.9198)						


**TETRAS ADL = β0 + β1·TETRAS-P + β2·HADS-D + β3·ECQ-ISE**.

### Reliability analysis

#### Patient cohort for the reliability analysis

Twenty-six of 93 patients were excluded from the reliability analyses because they were inadvertently given an older version of TETRAS PRO (6 patients), had treatment changes before day 30 (16 patients), or had incomplete data (4 patients). This 67-patient cohort did not differ statistically from the validity cohort in age, age of onset, duration of tremor, TETRAS ADL, TETRAS-P, and TETRAS PRO. Ten of the 67 patients (14.9%) were classified as ET plus, and the reasons for this classification were unsteady tandem gait (n = 7), body posturing that could be dystonic (n = 2), mild cognitive impairment (n = 3), and questionable or definite rest tremor (n = 4) (see Supplementary Material 4). No patient had less than 12 years of education. The validity and reliability cohorts had 50 patients in common.

#### Test-retest reliability

Repeated measures ANOVA revealed no statistically significant differences among the TETRAS PRO scores on day 0, day 1 and day 30 (F[49, 2] = 2.28, p = 0.108). The test-retest ICC for day 0 vs day 1 was 0.919 (95%CI 0.863–0.953), and the minimum detectable change (MDC95) was 7.2 points (95%CI 5.4–9.0).

The test-retest ICC for day 1 vs day 30 was 0.924 (95%CI 0.877–0.953), and the MDC95 for this time interval was 6.6 (95%CI 5.2–8.0). Fourteen patients reported a non-zero patient global impression of change (PGIC) on day 30, despite the absence of any change in treatment: 3 patients = –2, (worsening), 9 patients = –1, and 2 patients = +1 (improvement). The test-retest ICC with these patients excluded was 0.928 (95%CI 0.878–0.958), and the MDC95 was 6.6 (95%CI 5.0–8.2).

The median value of TETRAS-P was 22.5, which is comparable to that of other large clinic populations [3,20]. MDC95 for the lower half of our reliability cohort was 6.0 (95%CI 4.2–7.7) and 7.3 (95%CI 4.9–9.8) for the upper half.

Descriptive statistics and test-retest reliability of each item of TETRAS PRO are provided in Supplementary Material 4. The ICCs ranged from 0.670 (drinking item 4) to 0.926 (head item 2). The test-retest ICC for the leg item was 0.725.

## Discussion

The 14-item TETRAS PRO correlated strongly (r > 0.7) with the clinician-administered TETRAS-P subscale, the clinician-patient interview-based TETRAS ADL subscale, and the QUEST patient-reported quality of life scale. Of the 5 subscales of QUEST, TETRAS PRO correlated strongly with the physical subscale and weakly with the work/finances subscale. We conclude that TETRAS PRO has strong convergent validity.

We hypothesized that TETRAS PRO would be influenced by the patient’s mood and possibly by coping skills. We found a moderate correlation between TETRAS PRO and HADS-D and a weak correlation with HADS-A. TETRAS ADL but not TETRAS-P was also correlated with HADS-D. By contrast, both HADS-A and HADS-D were correlated with QUEST in our patients.

Previous studies have found a strong influence of depression on QUEST and on other patient reports of functional disability due to ET. A study of clinic-based and community-based patient cohorts in Germany found that tremor severity did not correlate with patient reports of ADL impairment, and only the Beck Depression Inventory score correlated with quality of life, as measured with the Short Form-12 questionnaire [21]. Similarly, clinical depression and anxiety were associated with greater self-reported disability and embarrassment from tremor, independent of tremor severity, in community-based and clinic-based cohorts from New York [22,23,24], and major depression and anxiety also influenced a performance-based test of tremor severity [22]. By contrast, we found no impact of depression or anxiety on our clinician assessment of patient performance, TETRAS-P. However, our multicenter clinic-based cohort had only one man (HADS-D = 13) and one woman (HADS-D = 17) with HADS-D greater than 8, so it is unlikely that more than two of our patients had major depression [31]. Only 7 patients had HADS-A greater than 10, the suggested cutoff for abnormal anxiety [25]. Furthermore, HADS has limited ability to distinguish between anxiety and depression [25]. Nevertheless, our results and those of previous studies indicate a need to control for depression in the interpretation of self-reported impairment of ADLs due to action tremor and to consider depressive symptoms when treating ET.

The Essen Coping Questionnaire information seeking and exchange of information subscale (ECQ-ISE) was the only ECQ subscale that was correlated with TETRAS PRO and TETRAS ADL. No subscale was correlated with TETRAS-P. The positive correlation with TETRAS PRO was small but suggests that patients who actively seek information and exchange experiences tend to rate their tremor higher. ECQ-ISE was not correlated with TETRAS-P, suggesting that ECQ-ISE was not driven by tremor severity. A study of clinic-based and community-based patient cohorts in Germany found that the main coping strategy in ET, measured with the Freiburg Questionnaire of Coping with Illness, was active problem-oriented coping, which is similar to ECQ-ISE [21].

TETRAS-P, HADS-D and ECQ-ISE combined to explain approximately 67% of the total variance of TETRAS PRO, with TETRAS-P being the principal driver, followed by HADS-D, and ECQ-ISE. TETRAS-P was not correlated with HADS or ECQ measures and may therefore be viewed as an assessment of tremor severity that is not influenced by psychosocial factors.

TETRAS PRO exhibited very good internal consistency (Cronbach alpha = 0.894 and McDonald omega = 0.903). All item-rest correlations exceeded 0.5 except for item 1 voice (0.386), item 2 head (0.064), and item 12 legs (0.356). These three items were viewed as unimportant by 8 or more patients that participated in the cognitive interviews for the initial construction of TETRAS PRO. We retained these items because they measured known features of ET (face validity). However, the TETRAS PRO leg item was only weakly correlated with the TETRAS-P leg item (rho = 0.186, p = 0.074), while the TETRAS PRO head and voice ratings correlated well with the corresponding TETRAS-P items (i.e., had good convergent validity). Twelve of 18 patients that underwent cognitive interviews considered the leg item of TETRAS PRO unimportant, and lower limb tremor is typically mild or absent in ET [26]. However, the leg item of TETRAS PRO had a test-retest reliability (ICC = 0.725) that was comparable to that of most other items in TETRAS PRO (Supplementary Material 4), and the inter-rater reliability of the leg item in TETRAS-P is poor [1]. Therefore, the value of the leg item in TETRAS PRO is questionable, but we found no compelling reason to discard it.

TETRAS PRO had excellent test-retest reliability over 30 days when no treatment change was in effect. The MDC 95%CI (MDC95) was 5.4–9.0 for a 1-day interval between assessments and 5.2–8.0 for a 30-day interval. Fourteen patients reported a positive or negative global impression of change at 30 days, despite no change in treatment. Test-retest reliability and MDC95 did not change significantly when these patients were excluded. Thus, the MDC95 is on average less than 0.5 point per item, which should be adequate to detect a clinically important change in ET.

There are limitations to our study. The participants were recruited from clinic populations at academic medical centers and were nearly all white and not Hispanic. Only two participants had less than a high school education. These findings may limit the generalizability of the TETRAS PRO in other patient populations. However, each item of TETRAS PRO and its five choices were carefully worded to achieve a Flesch-Kincaid readability grade level of 8 or less, and we used data from the cognitive interviews to ensure that each item of TETRAS PRO was consistently understood. The overall Flesch reading ease is 70.7, and the overall Flesch-Kincaid grade level is 6.7. With respect to race and ethnicity, there is some evidence to suggest that these may influence clinical expression of ET. For example, head tremor may be less common in Nigerians [27] and African Americans [28]. However, all people with ET or ET plus have upper limb tremor by definition [2], and 11 of 14 TETRAS PRO items are potentially influenced by upper limb tremor. Therefore, racial or ethnic differences in the clinical expression of ET or ET plus should not greatly impact the psychometrics of this scale.

Our use of ET plus may be viewed as a limitation because the distinction between ET and ET plus is purely definitional and highly subjective [29]. Only 9 of 67 patients in the validation cohort and 10 of 67 patients in the reliability cohort were classified as ET plus. We included this classification in our study design to encourage careful phenotyping of each patient and to hopefully reduce the enrollment of patients with other tremor syndromes, such as dystonia with tremor, Parkinson disease, and rhythmic cortical myoclonus.

Another limitation was that all 93 patients could not be included in the validity and reliability analyses due to protocol violations of using the wrong version of TETRAS PRO (slightly different wording of some items) on day 0 (26 patients) or on days 1 and 30 (6 patients), changes in treatment during the 30-day study period (16 patients), and missing data (4 patients). However, the main results of this study did not change significantly when all 93 patients were included. Specifically, the linear relationship of TETRAS PRO with TETRAS-P, HADS-D and ECQ-ISE had a coefficient of determination of 0.595, Cronbach alpha of TETRAS PRO was 0.874, and test-retest reliability was 0.878 (95%CI 0.820–0.918).

The anchors in TETRAS PRO emphasize whether tremor interferes with daily activities. This was intentional because TETRAS PRO was designed to assess the impact of treatment (or disease progression) in clinical trials and in routine clinical care. TETRAS PRO was not designed to identify modification strategies for reducing tremor, nor does it assess the effectiveness of such strategies. The anchors of most items are worded in a way that the use of such strategies elevates the item rating to at least 3.

Therefore, TETRAS PRO is a robustly valid and reliable measure of the impact of ET on ADLs and should be capable of detecting clinically important change. TETRAS PRO is the only ADL scale for ET that was developed in accordance with current best practices and guidelines for PROM development [6,8,30].

## Additional Files

The additional files for this article can be found as follows:

10.5334/tohm.875.s1 Supplementary Material 1. TETRAS PRO scale.

10.5334/tohm.875.s2 Supplementary Material 2. Results of cognitive interviews during the scale development phase.

10.5334/tohm.875.s3 Supplementary Material 3. McDonald omega, Cronbach alpha and item-rest correlation statistics, exploratory factor analysis and scree plot.

10.5334/tohm.875.s4 Supplementary Material 4. Demographics of the validity and reliability cohorts, Day 1 TETRAS PRO statistics, and Day 1 vs Day 30 test-retest ICC.
