# Supplementary material for: Validation of a New Patient-Reported Outcome Measure of the Functional Impact of Essential Tremor on Activities of Daily Living
Source: Tremor Other Hyperkinet Mov (N Y). 2024 May 14;14:26. doi: 10.5334/tohm.886 (PMC11100532; doi:10.5334/tohm.886)
Supplement: Supplementary Material 1. — TETRAS PRO scale. [file tohm-14-1-886-s1.pdf]

TETRAS PRO

Instructions: For each question, **circle or mark the answer that best describes your typical condition during the past week**. A family member or friend can circle your answers if you cannot do this yourself. However, we want you to answer each question without discussing the answers with other people.

1. Is your voice shaky or tremulous?

    0 = No. I have no tremor in my voice.

    1 = My voice tremor is so mild that I do not always notice it.

    2 = My voice is usually shaky, but I have no trouble speaking clearly.

    3 = My voice is so shaky that some words are difficult for people to understand.

    4 = My voice is so shaky that most words are difficult for people to understand.

2. Does your head shake?

    0 = No. I have no head tremor.

    1 = My head tremor is so mild that I do not always notice it.

    2 = My head is usually shaky, but this tremor is mild and does not bother me.

    3 = My head tremor bothers me, but it does not limit my daily activities.

    4 = My head tremor is so bad that it limits my ability to function.

3. Does tremor affect your ability to eat soup or other liquids with a regular soup spoon?

    0 = No. My spoon does not shake.

    1 = I am slightly shaky, but my tremor does not interfere with using a spoon.

    2 = My spoon shakes, but I usually do not spill anything, even when I use a regular spoon with one hand.

    3 = I am very shaky. I usually spill even when I use a special strategy, such as using two hands, using my less affected hand, or using a weighted spoon.

    4 = I cannot eat soup with any spoon because my tremor is so bad.

4. Does tremor affect your ability to drink liquids from a regular 8-ounce (250 ml) glass or cup?

    0 = No. I do not shake when I drink from a glass or cup.

    1 = I am slightly shaky, but my tremor does not interfere with drinking.

    2 = I am shaky, but I usually do not spill anything unless the glass or cup is very full.

    3 = I am very shaky. I sometimes spill even when I use a special strategy, such as using two hands, using the less affected hand, or drinking from a cup or glass that is not full.

    4 = I shake so much that I cannot drink from a regular glass or cup. I must use a straw or sippy cup.

5. Does tremor interfere with your hygiene or personal care (bathing, shaving, brushing teeth, using makeup)?

    0 = No. I do not notice tremor when performing my personal hygiene.

    1 = I am slightly shaky, but my tremor does not interfere with these tasks.

    2 = I have some difficulty due to tremor, but I am able to do everything without special strategies or equipment, such as using two hands, using the less affected hand, or using an electric razor.

    3 = I am unable to do some tasks such as putting on eye makeup or shaving unless I use a special strategy or equipment.

    4 = I shake so much that I cannot do one or more aspects of my personal hygiene without help from someone.

6. Does tremor interfere with your ability to dress yourself? This includes putting on jewelry, tying shoelaces, zipping zippers, and managing small buttons.

    0 = No. I notice no tremor when dressing.

    1 = Tremor is present when I dress, but it does not interfere with dressing.

    2 = Dressing is difficult due to tremor, but I have made no changes in what I wear or how I dress.

    3 = Tremor interferes with dressing. I have changed how I dress or what I wear, such as clothes with Velcro or no small buttons, and shoes with no laces.

    4 = My tremor is so bad that I cannot dress without help from someone.

7. Does tremor affect your ability to pour liquids from a carton, bottle or pitcher?

    0 = No. I notice no tremor when I pour liquids.

    1 = I am shaky, but my tremor does not interfere with pouring.

    2 = I have mild difficulty due to tremor. I can usually pour liquids without spilling and without using two hands or other strategies to avoid spilling.

    3 = Pouring liquids is very difficult. I spill too much unless I use two hands or other strategies to avoid spilling.

    4 = I no longer try to pour liquids because I spill too much from most cartons, bottles, or pitchers.

8. Does tremor affect your ability to pass food or drink to someone at your dinner table?

    0 = No. I notice no tremor when I do this.

    1 = I feel shaky, but I can pass any food or beverage container without spilling.

    2 = I can pass open containers of food and beverage if they are not full.

    3 = I cannot pass food or beverage unless it is in a closed container.

    4 = I shake too much to pass any food or drink, even in closed containers.

9. Does tremor limit your ability to use a keypad on a phone, lock or computer?

    0 = No. I notice no tremor when I use keypads.

    1 = I am shaky, but this does not interfere with my ability to use most keypads.

    2 = My tremor interferes with the use of most keypads, but I still manage with one hand.

    3 = I can use keypads if they are large enough, but I must use two hands or brace my hand is some way.

    4 = I shake too much to use any keypad.

10. Does tremor affect your ability to write?

    0 = No. My hands do not shake when I write.

    1 = My hands are sometimes shaky, but little or no tremor is visible in my handwriting.

    2 = My handwriting is shaky, but people can usually read what I write.

    3 = People usually cannot read some words that I write because my handwriting is so shaky.

    4 = My tremor is so bad that I cannot write legibly, even when I use two hands.

11. Does tremor affect your job performance? This would include being a homemaker. If you are retired, consider how your current tremor would have affected your previous job.

 0 = No. Tremor has no effect on my job performance.

 1 = Tremor makes some tasks more difficult, but most aspects of my job are not affected by tremor.

 2 = Most aspects of my job are more difficult because of tremor, but I am still able to do all of my responsibilities.

 3 = Tremor limits what job I can do. I could not continue my job without a change in responsibilities or without using special equipment.

 4 = I cannot do any job because of tremor.

12. Do you notice tremor in your legs?

 0 = No. I never notice tremor in my legs.

 1 = Yes, my legs sometimes feel slightly shaky, but this shakiness is not visible and does not affect the use of my legs.

 2 = My legs feel shaky, but my tremor is barely visible. I am able to do everything normally, but my shakiness can be uncomfortable or make me feel unsteady.

 3 = My legs are visibly shaky when I am standing or when I try to hold my legs steady in one position. I am able to do everything, but my leg tremor makes some activities difficult.

 4 = My tremor is so severe that I am unsteady when standing or walking.

13. Think about how tremor affects your daily activities. In the space below, please write the activity that is most important to you.

Activity ________________________________

How much does your tremor interfere with this activity?

 0 = Not at all. I do not notice tremor when I perform this activity.

 1 = Tremor is present, but it does not affect my performance.

 2 = Tremor makes this activity more difficult, but I am still able to do it without using special strategies or aids.

 3 = My tremor is so bad that I cannot do this activity unless I use a special strategy or aid.

 4 = I cannot do this activity because my tremor is too severe.

14. Has tremor affected your social activities?

0 = No. I do not think about tremor when I am out socially.

1 = I am conscious of tremor, but I still enjoy my social activities.

2 = My social activities have not changed, but they are less enjoyable due to tremor.

3 = I have stopped some social activities because of tremor.

4 = My tremor is so bad that I try to avoid all social activities.

Please identify anyone who assisted the patient in completing this questionnaire (e.g., spouse, friend):

0 = No one helped me

1 = spouse

2 = other family member

3 = friend

4 = caregiver

5 = other person
