# Supplementary material for: Validation of a New Patient-Reported Outcome Measure of the Functional Impact of Essential Tremor on Activities of Daily Living
Source: Tremor Other Hyperkinet Mov (N Y). 2024 May 14;14:26. doi: 10.5334/tohm.886 (PMC11100532; doi:10.5334/tohm.886)
Supplement: Supplementary Material 2. — Results of cognitive interviews during the scale development phase. [file tohm-14-1-886-s2.pdf]

**Table 1. Responses to the cognitive interview question: "Is the question clear?"**

| ***Item #*** | **Yes** | **No** |
|---|---|---|
| 1. voice | 18 | 0 |
| 2. head | 18 | 0 |
| 3. spoon | 17 | 1* |
| 4. drinking | 17 | 1* |
| 5. hygiene | 18 | 0 |
| 6. dressing | 18 | 0 |
| 7. pouring | 18 | 0 |
| 8. carrying | 18 | 0 |
| 9. keypad | 18 | 0 |
| 10. writing | 18 | 0 |
| 11. work | 18 | 0 |
| 12. legs | 18 | 0 |
| 13. task | 18 | 0 |
| 14. social | 18 | 0 |

* The NO answers came from the same patient. Wanted greater precision in the wording (e.g., size of cup).

**Table 2. Responses to questions regarding clarity of potential TETRAS PRO response choices**

"Are the five possible choices clear? Did you have trouble answering this question?"

| | **Choices clear?** | | ***Trouble answering?*** | |
|---|---|---|---|---|
| ***Item #*** | **Yes** | **No** | **Yes** | **No** |
| 1. voice | 18 | 0 | 2** | 16 |
| 2. head | 18 | 0 | 0 | 18 |
| 3. spoon | 17 | 1* | 1** | 17 |
| 4. drinking | 18 | 0 | 1** | 17 |
| 5. hygiene | 18 | 0 | 1** | 17 |
| 6. dressing | 18 | 0 | 0 | 18 |
| 7. pouring | 17 | 1* | 1* | 17 |
| 8. carrying | 18 | 0 | 1** | 17 |
| 9. keypad | 18 | 0 | 1*** | 17 |
| 10. writing | 18 | 0 | 2** | 16 |
| 11. work | 18 | 0 | 1** | 17 |
| 12. legs | 18 | 0 | 0 | 18 |
| 13. task | 17 | 1** | 4** | 14 |
| 14. social | 18 | 0 | 1* | 17 |

* Size of pitcher? More precision or clarification required.
** Trouble deciding between two choices due to individual circumstances.
*** No longer uses a key.

**Table 3.** **Distributions of TETRAS PRO ratings for item and classification of importance.**

| ***TETRAS PRO item*** | | **Rating** | | | | |
|---|---|---|---|---|---|---|
| ***Item #*** | **Important?** | **0** | **1** | **2** | **3** | **4** |
| *1* | no | **** | **** | ** | | |
| *voice* | yes | **** | * | * | ** | |
| *2* | no | **** | *** | | | * |
| *head* | yes | ** | | *** | **** | * |
| *3* | no | * | | ** | | |
| *spoon* | yes | | *** | *** | ****** | *** |
| *4* | no | | ** | | | |
| *drinking* | yes | * | **** | ** | ****** | *** |
| *5* | no | **** | | ** | | |
| *hygiene* | yes | * | * | * | ******* | ** |
| *6* | no | ***** | ***** | | | |
| *dressing* | yes | ** | * | ** | *** | |
| *7* | no | | **** | | * | |
| *pouring* | yes | * | *** | * | **** | **** |
| *8* | no | | ** | * | * | |
| *carrying* | yes | * | | ******* | *** | *** |
| *9* | no | ** | **** | | *** | |
| *keypad* | yes | * | | ***** | *** | |
| *10* | no | | | * | | |
| *writing* | yes | * | | ******** | ****** | ** |
| *11* | no | | ** | * | | |
| *work* | yes | ** | * | **** | *** | ***** |
| *12* | no | ***** | ***** | ** | | |
| *legs* | yes | ** | ** | | ** | |
| *13* | no | | | * | | |
| *task* | yes | * | | ***** | ****** | ***** |
| *14* | no | * | **** | ** | | |
| *social* | yes | * | **** | * | * | **** |

**Additional description of scale development phase following the cognitive interviews**

The structured interviews also included the question "Is there something important about your tremor that is not addressed with the questions in the questionnaire that you completed?" All participants responded NO.

Data from the structured interviews dictated some rewording of the items but no substantive changes in TETRAS PRO version 2. TETRAS PRO version 2 was then given to the other movement disorder specialists in this study (N = 8) for their comments on relevance and comprehensiveness. This resulted in some changes in item wording but no addition or deletion of items (TETRAS PRO version 3). Thus, this structured interview process addressed each element of the COSMIN guideline for content validity: 2a) Asking patients about relevance, 2b) Asking patients about comprehensiveness, 2c) Asking patients about comprehensibility, 2d) Asking professionals about relevance, and 2e) Asking professionals about comprehensiveness.
