# Supplementary material for: Validation of a New Patient-Reported Outcome Measure of the Functional Impact of Essential Tremor on Activities of Daily Living
Source: Tremor Other Hyperkinet Mov (N Y). 2024 May 14;14:26. doi: 10.5334/tohm.886 (PMC11100532; doi:10.5334/tohm.886)

Table 1. McDonald omega, Cronbach alpha and item-rest correlation values for TETRAS PRO

	If item dropped		
**TETRAS PRO item**	**McDonald ω**	**Cronbach α**	**Item-rest correlation**
tetras_pro_voice	0.903	0.894	0.386
tetras_pro_head	0.914	0.909	0.064
tetras_pro_eating	0.890	0.881	0.717
tetras_pro_drinking	0.889	0.879	0.743
tetras_pro_hygiene	0.893	0.884	0.646
tetras_pro_dressing	0.891	0.882	0.688
tetras_pro_pouring	0.891	0.881	0.707
tetras_pro_carrying	0.891	0.881	0.722
tetras_pro_keypad	0.889	0.880	0.754
tetras_pro_writing	0.899	0.889	0.527
tetras_pro_working	0.893	0.883	0.662
tetras_pro_legs	0.905	0.896	0.356
tetras_pro_task	0.897	0.888	0.570
tetras_pro_social	0.896	0.886	0.597

Table 2. Exploratory factor analysis

Chi-squared Test:

	Value	df	p
Model	75.554	77	0.525

Factor loadings:

TETRAS PRO ITEM	FACTOR 1	UNIQUENESS
tetras_pro_drinking	0.805	0.351
tetras_pro_keypad	0.783	0.387
tetras_pro_carrying	0.778	0.394
tetras_pro_pouring	0.762	0.419
tetras_pro_eating	0.754	0.432
tetras_pro_dressing	0.734	0.461
tetras_pro_hygiene	0.698	0.513
tetras_pro_working	0.690	0.524
tetras_pro_social	0.638	0.593
tetras_pro_task	0.602	0.637
tetras_pro_writing	0.544	0.704
tetras_pro_voice	0.377	0.858
tetras_pro_head	0.364	0.997
tetras_pro_legs	0.059	0.868

Note: Applied rotation method is Promax

Figure. Scree plot for TETRAS PRO

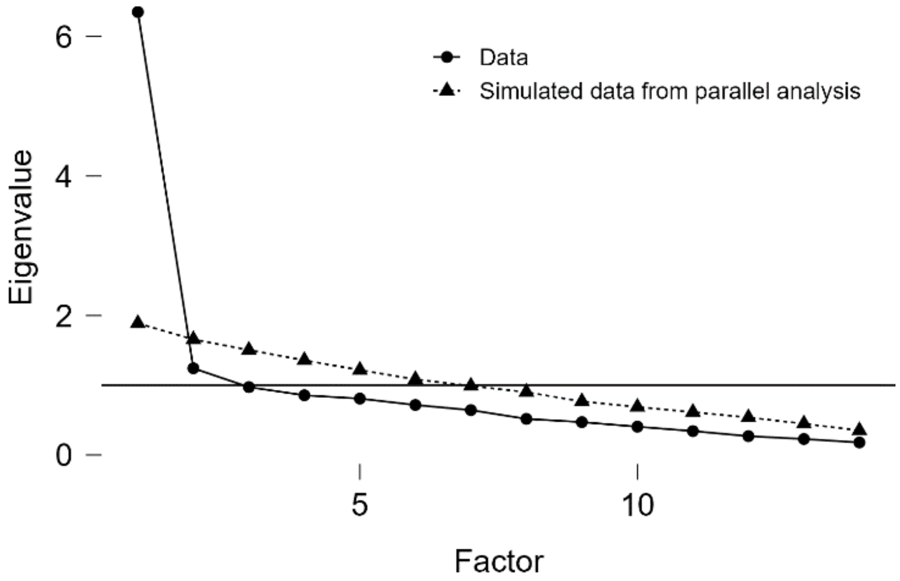

Supplement: Supplementary Material 3. — McDonald omega, Cronbach alpha and item-rest correlation statistics, exploratory factor analysis and scree plot. [file tohm-14-1-886-s3.pdf]
