# Supplementary material for: Validation of a New Patient-Reported Outcome Measure of the Functional Impact of Essential Tremor on Activities of Daily Living
Source: Tremor Other Hyperkinet Mov (N Y). 2024 May 14;14:26. doi: 10.5334/tohm.886 (PMC11100532; doi:10.5334/tohm.886)
Supplement: Supplementary Material 4. — Demographics of the validity and reliability cohorts, Day 1 TETRAS PRO statistics, and Day 1 vs Day 30 test-retest ICC. [file tohm-14-1-886-s4.pdf]

Patient cohort for validity analyses

	Age		Age of onset		Duration of tremor	
gender	0 (male)	1	0	1	0	1
N	37	30	37	30	37	30
Minimum	31.8	42.3	5.0	7.0	4.6	3.8
Maximum	81.0	82.6	74.0	70.0	67.0	67.4
Mean	67.4	69.1	37.6	42.7	29.8	26.4
95% CI	63.8 to 71.0	65.8 to 72.4	31.0 to 44.1	35.3 to 50.0	23.4 to 36.3	19.6 to 33.3
Median	69.3	71.3	40.0	45.5	24.2	20.5
95% CI	65.9 to 73.5	67.9 to 73.4	30.0 to 50.0	35.5 to 56.7	16.9 to 33.2	13.6 to 35.3
SD	10.9	8.8	19.6	19.8	19.3	18.4
25 - 75 percentile	62.8 to 75.8	65.5 to 75.1	19.3 to 52.8	27.0 to 60.0	13.6 to 48.0	12.6 to 38.5
Normal Distribution*	0.0002	0.02	0.08	0.04	0.006	0.008

* Shapiro-Wilk test (p value)

	TETRAS ADL		TETRAS Performance		TETRAS PRO	
gender	0 (male)	1	0	1	0	1
N	37	30	37	30	37	30
Minimum	5	13	7.5	7.0	6	4
Maximum	39	40	36.0	43.0	37	49
Mean	24.3	24.3	23.5	22.0	22.2	23.6
95% CI	21.8 to 26.9	21.8 to 26.8	21.4 to 25.6	19.2 to 24.8	19.5 to 25.0	19.7 to 27.5
Median	26	24	24.5	20.8	23	24
95% CI	20 to 30	21 to 27	22.0 to 26.0	19.0 to 22.9	17 to 27	20 to 27
SD	7.6	6.7	6.2	7.6	8.2	10.4
25 - 75 percentile	18 to 31	19 to 29	20.8 to 27.1	18.5 to 28.0	16 to 28	16 to 31
Normal Distribution*	0.35	0.81	0.65	0.39	0.49	0.92

* Shapiro-Wilk test (p value)

	Age	Age of onset	Duration of tremor	TETRAS ADL	TETRAS Performance	TETRAS PRO
N	67	67	67	67	67	67
Minimum	31.8	5.0	3.8	5	7.0	4
Maximum	82.6	74.0	67.4	40	43.0	49
Mean	68.2	40.0	28.3	24.3	22.8	22.9
95% CI	65.7 to 70.6	35.1 to 44.6	23.7 to 32.9	22.6 to 26.1	21.1 to 24.5	20.6 to 25.1
Median	70.7	42.0	23.9	24	22.5	23
95% CI	67.7 to 72.2	35.0 to 50.0	17.1 to 28.9	22 to 27	21.0 to 25.0	21 to 26
SD	10.0	19.7	18.8	7.2	6.9	9.2
25 - 75 percentile	64.175 to 75.325	20.500 to 55.000	13.150 to 41.425	18 to 30	19.0 to 27.4	16 to 28
Normal Distribution*	<0.0001	0.0036	0.0001	0.59	0.87	0.73

* Shapiro-Wilk test (p value)

Frequency tables

Variable<br>0 = male<br>1 = female	gender

0	37	55.2%
1	30	44.8%
Total	67	100.0%

Variable<br>0 = not Hispanic<br>1 = Hispanic	ethnicity

0	63	94.0%
1	4	6.0%
Total	67	100.0%

Variable<br>1 = right<br>2 = left	handedness

1	59	88.1%
2	8	11.9%
Total	67	100.0%

Variable<br>2 = Asian<br>3 = Black<br>5 = White	race

2	1	1.5%
3	2	3.0%
5	64	95.5%

Total	67	100.0%

Variable<br>1 = ET<br>2 = ET plus	diagnosis

1	58	86.6%
2	9	13.4%
Total	67	100.0%

Variable	Education (years)

6	1	1.5%
11	1	1.5%
12	12	17.9%
13	7	10.4%
14	8	11.9%
15	1	1.5%
16	15	22.4%
17	3	4.5%
18	9	13.4%
19	2	3.0%
20	7	10.4%
24	1	1.5%
Total	67	100.0%

Reported clinical signs in patients with ET plus (Validity Cohort)

Patient	Unsteady tandem gait	Unusual posturing	questionable rest tremor	jerky tremor	peripheral neuropathy	mild cognitive impairment	rest tremor
5	X	X					
12	X						
13						X	
15	X						
16						X	X
3250-1							X
3253-3		X					
3253-7	X					X	
3253-8	X	X					

Patient cohort for reliability analyses

	Age		Age of onset		Duration of tremor	
gender	0 (male)	1	0	1	0	1
N	33	34	33	34	33	34
Minimum	31.8	46.1	8.0	6.0	2.7	3.8
Maximum	86.9	83.0	81.0	70.0	67.0	68.7
Mean	69.2	70.8	41.5	46.3	27.7	24.5
95% CI	65.6 to 72.8	67.8 to 73.7	34.1 to 48.8	39.5 to 53.1	20.5 to 34.9	17.9 to 31.1
Median	70.7	72.9	47.0	51.0	23.1	17.1
95% CI	67.5 to 74.5	69.6 to 75.4	31.6 to 51.0	41.7 to 60.0	14.5 to 29.9	13.0 to 26.4
SD	10.2	8.5	20.7	19.6	20.3	19.0
25 - 75 percentile	64.3 to 76.0	67.0 to 75.7	20.0 to 55.5	38.0 to 62.0	11.5 to 47.3	12.6 to 36.6
Normal Distribution*	0.0044	0.014	0.13	0.0032	0.0024	0.0002

* Shapiro-Wilk test (p value)

	TETRAS ADL		TETRAS Performance		TETRAS PRO	
gender	0 (male)	1	0	1	0	1
N	33	34	33	34	33	34
Minimum	5	4	7.5	7.0	2	5
Maximum	35	35	36.0	31.0	38	43
Mean	22.8	23.1	22.5	19.9	20.3	21.6
95% CI	20.1 to 25.4	20.6 to 25.5	20.5 to 24.6	17.7 to 22.1	17.2 to 23.5	18.4 to 24.7
Median	22	24	23.5	20.0	23	23
95% CI	20 to 27	20 to 27	21.2 to 25.0	17.2 to 21.6	16 to 26	19 to 27
SD	7.5	6.9	5.8	6.2	8.8	9.1
25 - 75 percentile	17 to 29	18 to 28	19.8 to 26.0	15.0 to 25.0	16 to 27	14 to 29
Normal Distribution*	0.25	0.62	0.37	0.75	0.24	0.33

* Shapiro-Wilk test (p value)

	Age	Age of onset	Duration of tremor	TETRAS ADL	TETRAS Performance	TETRAS PRO
N	67	67	67	67	67	67
Minimum	31.8	6.0	2.7	4	7.0	2
Maximum	86.9	81.0	68.7	35	36.0	43
Mean	70.0	43.9	26.1	22.9	21.2	21.0
95% CI	67.7 to 72.3	39.0 to 48.8	21.3 to 30.8	21.2 to 24.7	19.7 to 22.7	18.8 to 23.1
Median	72.1	48.0	19.1	23	21.500	23
95% CI	69.3 to 74.1	42.0 to 53.0	15.1 to 25.9	20 to 26	20.0 to 23.5	19 to 25

SD	9.3	20.2	19.5	7.2	6.1	8.9
25 - 75 percentile	65.5 to 75.9	30.0 to 60.0	12.0 to 37.7	18 to 28	17.1 to 25.5	14 to 27
Normal Distribution*	0.0002	0.0021	<0.0001	0.13	0.60	0.12

* Shapiro-Wilk test (p value)

Frequency tables

Variable	gender
0 = male<br>1 = female	

0	33	49.3%
1	34	50.7%
Total	67	100.0%

Variable	ethnicity
0 = not Hispanic<br>1 = Hispanic	

0	64	95.5%
1	3	4.5%
Total	67	100.0%

Variable	handedness
1 = right<br>2 = left	

1	59	89.4%
2	7	10.6%
Total	66	100.0%

Variable	race
Filter	

2	1	1.5%
3	1	1.5%
5	65	97.0%
Total	67	100.0%

Variable	diagnosis
1 = ET<br>2 = ET plus	

1	57	85.1%
2	10	14.9%
Total	67	100.0%

Variable	Education (years)

12	10	14.9%
13	6	9.0%
14	8	11.9%
15	1	1.5%

16	16	23.9%
17	4	6.0%
18	11	16.4%
19	3	4.5%
20	7	10.4%
24	1	1.5%
Total	67	100.0%

Reported clinical signs in patients with ET plus (Reliability Cohort)

Patient	Unsteady tandem gait	Unusual posturing	questionable rest tremor	jerky tremor	peripheral neuropathy	mild cognitive impairment	rest tremor
5	X	X					
12	X						
13						X	
16						X	X
3250-1							X
3253-8	X	X					
3255-5	X		X				
3255-6	X						
3255-8	X		X				
3255-11	X					X	

Day 1 TETRAS PRO statistics, and Day 1 vs Day 30 test-retest ICC

TETRAS PRO items	N	Minimum	Maximum	Mean	95% CI	Median	95% CI	25 - 75 %tile	Test-retest ICC
Item 1 voice	67	0	4	0.6	0.4 to 0.8	0	0 to 1	0 to 1	0.857
Item 2 head	67	0	3	0.9	0.6 to 1.2	0	0 to 1	0 to 2	0.926
Item 3 eating	67	0	4	2.2	2.0 to 2.5	3	2 to 3	2 to 3	0.827
Item 4 drinking	67	0	4	1.9	1.7 to 2.2	2	2 to 2	1 to 3	0.670
Item 5 hygiene	67	0	4	1.6	1.3 to 1.8	2	1 to 2	0 to 2	0.818
Item 6 dressing	67	0	3	1.2	0.9 to 1.4	1	1 to 1	0 to 2	0.778
Item 7 pouring	67	0	4	2.0	1.8 to 2.2	2	2 to 3	1 to 3	0.843
Item 8 carrying	67	0	4	1.8	1.5 to 2.0	2	1 to 2	1 to 2	0.776
Item 9 keypad	67	0	3	1.5	1.3 to 1.7	1	1 to 2	1 to 2	0.743
Item 10 writing	67	0	4	2.3	2.1 to 2.5	2	2 to 3	2 to 3	0.842
Item 11 working	67	0	4	1.5	1.2 to 1.7	1	1 to 2	1 to 2	0.872
Item 12 legs	67	0	2	0.3	0.2 to 0.5	0	0 to 0	0 to 0	0.725
Item 13 task	67	0	4	2.2	2.0 to 2.5	2	2 to 3	2 to 3	0.777
Item 14 social	67	0	4	0.9	0.7 to 1.2	1	1 to 1	0 to 1	0.745
